# Experimental Models of Early Exposure to Alcohol: A Way to Unravel the Neurobiology of Mental Retardation

**DOI:** 10.3389/fped.2014.00142

**Published:** 2015-01-06

**Authors:** Alberto Granato, Andrea De Giorgio

**Affiliations:** ^1^Department of Psychology, Catholic University of the Sacred Heart, Milan, Italy

**Keywords:** fetal alcohol spectrum disorders, development, cerebral cortex, amygdala, apoptosis, intellectual disability, glial cells, epigenetics

As of November 2014, a PubMed search for “fetal alcohol” retrieved more than 14,500 articles. Alcohol consumption during pregnancy and its detrimental consequences on the developing brain raise major public health, social, and economic issues. However, the research on fetal alcohol spectrum disorders (FASD) in the real world is challenging, given that it is largely based on retrospective analysis. Therefore, establishing the relationship between brain damage and drinking habits proves particularly hard. One of the advantages of FASD studies carried out in the laboratory environment derives from the tight control of time, dose, and modality of alcohol exposure (1). Furthermore, since FASD are among the leading causes of intellectual disability, animal models of early exposure to alcohol represent an invaluable tool to elucidate the basic neurobiological mechanisms leading to the cognitive defects. Experimental models of genetic syndromes are ideally suited to study the role of single molecules, such as the fragile X mental retardation protein, throughout the maturation of the nervous system. Conversely, experimental exposure to alcohol can be carried out during discrete, often very restricted, time windows and, though depending on the interference with several molecular pathways, can provide information about which developmental periods and brain areas are critically involved in the genesis of the intellectual disability.

In the present Research Topic, hosted by Frontiers in Pediatrics, we have gathered some of the most outstanding scientists, among those actively involved in the experimental study of FASD. The reader will be browsing through different subfields of basic research on FASD and we are confident that he/she will get a comprehensive view of the topic, including open questions and useful hints for novel therapeutic interventions.

The review article by Brian Christie and coworkers provides a useful guide for the experimental neurobiologist, highlighting pros and cons of the most widely used animal models of FASD (2). In this paper, there is a particular focus on how to study the behavioral consequences of developmental alcohol exposure. Antonella Peruffo and Bruno Cozzi point out that *in vitro* experiments dealing with neurodegenerative disorders or FASD can be carried out on primary cultures from the fetal bovine brain (3). In view of the concerns raised by the Institutions and by the general public on animal experimentation, species already used for alimentary purposes represent a valuable alternative.

Alcohol can interfere with cell populations that pave the way for brain development. The interaction of ethanol with the pioneering cortical preplate is described in the review by Eric Olson, where a strong focus is devoted to gene expression during early cortical development (4). Neuroscientists sometimes forget the role of glial cells. Thus, we are grateful to Marina Guizzetti and her coworkers, who shed light on how glial cells guide neural development and how this pivotal function can be disrupted during FASD (5).

There is no doubt that the cerebral cortex is one of the key structures affected by early exposure to alcohol and its impairment is responsible for most of the cognitive defects observed in FASD. Alexandre Medina and coworkers contributed to this Research Topic with an original research article in which, combining different methods, they describe deep alterations affecting the visual cortex and the visual pathways of mice exposed to alcohol during early postnatal life (6). Such anomalies can result from the disruption of visual cortical plasticity, demonstrated in previous works from the same lab. In our mini-review in Ref. (7), we focus on neocortical pyramidal neurons and show that FASD and other types of mental retardation are characterized by several, often contrasting, alterations of this heterogeneous neuron population.

Although the cortex and cerebellum are the most studied structures among those damaged by the early effects of alcohol, other brain areas appear to be affected as well. Surprisingly, the amygdala, despite its key role in the emotional and social life, has received little attention. Two original research articles, hosted in this Research Topic, deal with amygdalar alterations in experimental FASD. Using a combination of biochemical, electrophysiological, and behavioral techniques, Fernando Valenzuela and coworkers demonstrated an impairment of dopamine-regulated GABA neurotransmission in the basolateral amygdala (8). Cherry Ignacio, Sandra Mooney, and Frank Middleton (9) studied the micro-RNA expression in the amygdala of rats prenatally exposed to ethanol. It is worth mentioning that the observed alterations were partially reversed by social enrichment. Needless to say, both these papers, focused on the amygdala, display a great potential for the discovery of new therapeutic interventions.

Recently, the gap between genetic and environmental influence has been bridged by the advent of a discipline usually referred to as epigenetics. In their comprehensive review, Shiva Singh and coworkers point out that epigenetic mechanisms (such as those related to DNA methylation) give a substantial contribution to the genesis of the intellectual disability observed in FASD (10). We guess that the epigenetics of FASD will open new, exciting possibilities for the interpretation of syndromes featuring mental retardation.

Finally, John Olney, in his opinion article (11), raises a challenging issue: even though FASD alterations are manifold, yet all of them can be reconducted to a single starting point, namely the apoptosis. Therefore, most of the research efforts should be concentrated on the treatment of the widespread, ethanol-induced neuronal death. This line of reasoning can be further extended to several conditions, such as the effects of other drugs of abuse and/or of largely used anesthetics.

In conclusion, we wish to thank all the neuroscientists who gave their valuable contribution to this Research Topic. We are confident that their commitment to the experimental work on FASD will ultimately result in a great improvement of our ability to understand the intellectual disability.

## Conflict of Interest Statement

The authors declare that the research was conducted in the absence of any commercial or financial relationships that could be construed as a potential conflict of interest.
